# Sick Sinus Syndrome and Takotsubo Cardiomyopathy

**DOI:** 10.1155/2018/3868091

**Published:** 2018-08-19

**Authors:** Ahmed S. Yassin, Ahmed Subahi, Hossam Abubakar, Ahmed Rashed, Mohamed Shokr

**Affiliations:** ^1^Department of Internal Medicine, Wayne State University/Detroit Medical Center, Detroit, MI, USA; ^2^Division of Cardiovascular Medicine, Department of Internal Medicine, Wayne State University/Detroit Medical Center, Detroit, MI, USA

## Abstract

*Background*. Takotsubo cardiomyopathy is associated with increased risk of ventricular arrhythmias, atrial fibrillation, and bradyarrhythmias. However, sinus node dysfunction is relatively infrequent in the setting of takotsubo cardiomyopathy. *Case Report*. We are reporting a case of a 73-year-old woman with a history of asymptomatic sinus bradycardia who developed sick sinus syndrome complicated by takotsubo cardiomyopathy. *Conclusion*. Acute symptomatic sick sinus syndrome in patients with preexisting silent sinus node dysfunction can trigger takotsubo cardiomyopathy. Understanding precipitating factors of takotsubo cardiomyopathy and identifying the patients at risk of life-threatening arrhythmia can help in refining risk stratification and therapy planning. Patients with sick sinus syndrome complicated by takotsubo cardiomyopathy may benefit from pacemaker implantation. However, evaluation on a case-by-case basis is mandatory.

## 1. Introduction

First described in Japan by Dote et al. in 1990, takotsubo cardiomyopathy (TC) became increasingly reported as a form of reversible focal myocardial hypokinesis following significant psychological or physical stress [1, 2]. Arrhythmias are common in patients with TC including atrial fibrillation (5–15% of the cases) and ventricular arrhythmias (4–9% of the cases) [2]. However, sinus node dysfunction (SND) is relatively infrequent in the setting of TC (1.3% of the cases) [3]. We describe the case of a 73-year-old woman with a history of asymptomatic sinus bradycardia who developed sick sinus syndrome (SSS) complicated by takotsubo cardiomyopathy.

## 2. Case Presentation

**Figure 1:**
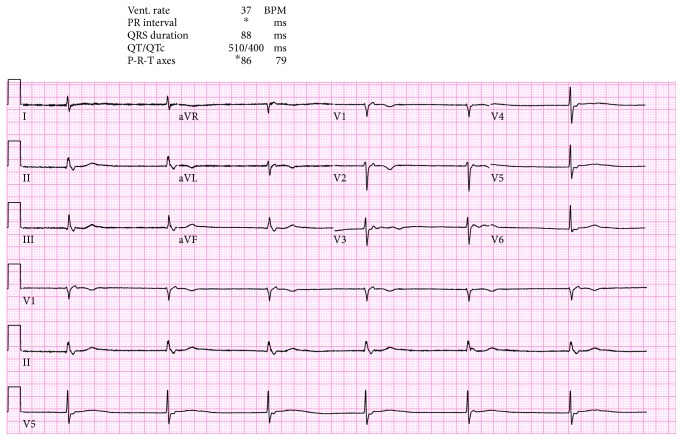
EKG shows junctional escape rhythm with a heart rate (HR) of 37 beats/minute.

**Figure 2:**
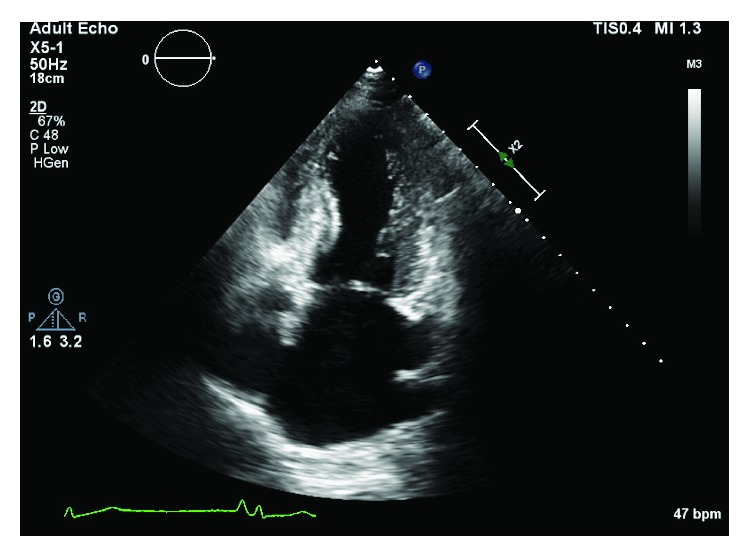
Echocardiogram (2-chamber long-axis view) showing the apical ballooning suggestive of takotsubo cardiomyopathy.

**Figure 3:**
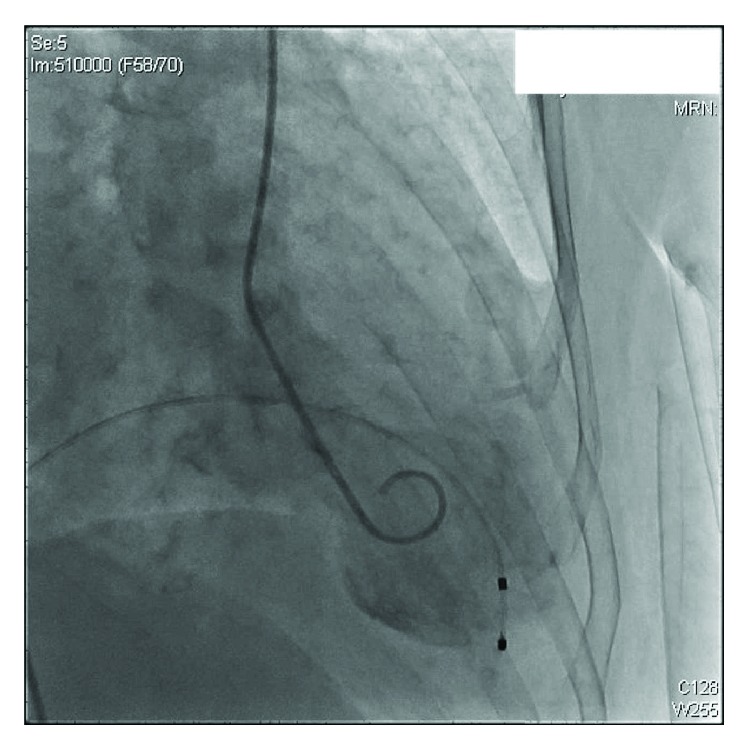
Left ventriculogram in the right anterior oblique projection showing the ventricular ballooning suggestive of takotsubo cardiomyopathy.

**Figure 4:**
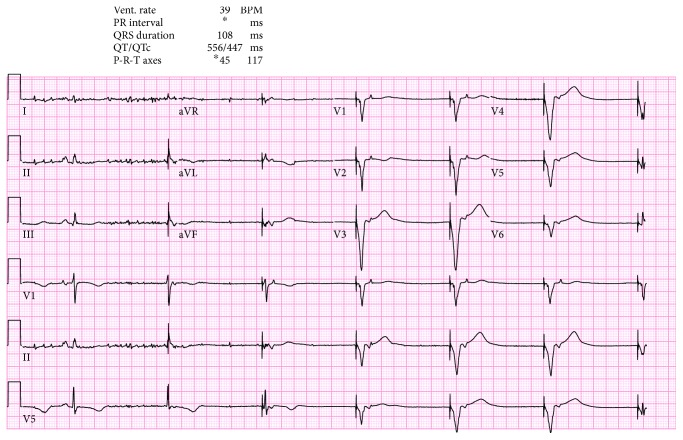
EKG shows one sinus beat followed by several ventricular paced beats.

**Figure 5:**
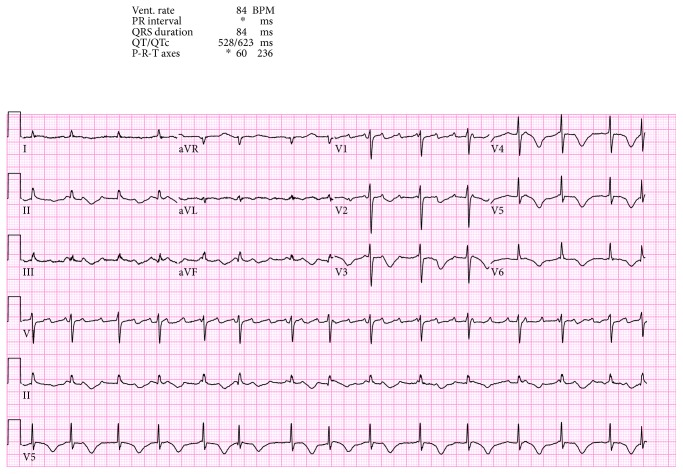
EKG shows atrial flutter with variable AV block.

We present the case of a 73-year-old woman with a history of asymptomatic sinus bradycardia that presented to our facility with a chief complaint of acute chest pain. She was found to have elevated troponin I of 0.3 ng/ml (normal < 0.057). Chest X-ray was normal. Laboratory workup revealed normal complete blood count, bleeding profile, basic metabolic panel, thyroid-stimulating hormone, and random blood glucose. The 12-lead electrocardiogram (ECG) was suggestive of junctional escape rhythm with a heart rate (HR) of 37 beats/minute (Figure 1). Transthoracic echocardiography (TTE) showed reduced left ventricular function (ejection fraction (EF) of 30%), with apical ballooning suggestive of takotsubo cardiomyopathy versus acute coronary syndrome (Figure 2). Left ventriculogram also showed ventricular ballooning suggestive of takotsubo cardiomyopathy (Figure 3). Cardiac catheterization revealed normal coronary arteries. Thus, she was diagnosed with classical TC. Due to her symptomatic bradycardia, a temporary pacing wire was placed. Her diagnosis was suggestive of sick sinus syndrome as she had occasional P waves that were fine (Figure 4). Overnight, she went into an atrial flutter with a variable block (Figure 5). Although, no rate control agent was started, the HR was 83 beats/minute (Figure 5). Her atrial flutter continued over the next 24 hours. A dual-chamber pacemaker was recommended and placed without complications by the electrophysiology team. Her rhythm on discharge was rate-controlled atrial flutter. She was discharged home on a beta-blocker, angiotensin-converting enzyme inhibitor, and anticoagulation. Her TTE findings were resolved on a follow-up visit two weeks later. However, pacemaker interrogation revealed frequent episodes of paroxysmal rate-controlled atrial fibrillation alternating with bradycardia requiring cardiac pacing. The patient is still under regular follow-up in the cardiology clinic.

## 3. Discussion

Takotsubo cardiomyopathy is characterized by reversible regional dyskinesia of the ventricular myocardium typically extending beyond a single coronary vascular bed [2]. TC can mimic acute heart failure or acute myocardial infarction in clinical presentation and be usually but not always triggered by medically or emotionally stressful events [2, 4]. The absence of significant coronary stenosis is a prerequisite for the diagnosis on almost all accepted diagnostic criteria [2, 4]. Arrhythmia is a common complication in patients with TC [2]. Also, TC can evolve after discrete episodes of arrhythmia [3]. However, SND in the setting of TC is relatively infrequent. In a review by Syed et al., new SND was observed in 11 of 816 TC patients (1.3%) [3]. Tsuchihashi et al. in a case series of 88 patients with TC reported sinus bradycardia in nine patients [5]. Dib et al. reported sinus pauses (up to 7.4 seconds) in one patient who eventually developed asystole mandating temporary pacemaker and inotropic support [6]. Hertting et al. reported one patient with symptomatic SSS requiring permanent pacemaker implantation [7]. Also, a case report by Kim described symptomatic SSS during the index episode requiring permanent pacemaker implantation [8]. However, in the previously reported cases, it is unclear whether TC is the cause or effect of SND. Theoretically, TC can cause SND through several mechanisms [3, 9]. Adrenergic compensative activation following bradycardia can induce transient myocardial dysfunction [10]. Ueyama et al. reported left ventriculography normalization in emotionally stressed rats with induced reversible LV apical ballooning, by pretreatment with adrenal receptor blockade [11, 12]. In time, catecholamine stress in TC can lead to a secondary increase in vagal tone with subsequent SND [3], and catecholamine-mediated myocardial stunning was considered crucial in the pathogenesis of TC. Therefore, reflex sympathetic stimulation to counteract severe SND may trigger TC. Age-related sinus node degeneration may also exacerbate that effect.

However, our patient had preexisting evidence of SND. She was seen in the cardiology outpatient clinic two months prior to admission for asymptomatic bradycardia. She was not on any negative chronotropic medication. Her EKG showed sinus bradycardia with no PR prolongation or conduction block. A 48 hr Holter monitor showed slow heart rate (HR) during sleep (30–40 beats/min) with an early morning pause of 3.5 seconds and an average HR of 61 beats/min. TTE showed EF 55–60%, with no regional wall motion abnormality. Thus, no intervention was necessary at that time. On admission emotional stress, medical stress and the intake of negative chronotropic drugs were ruled out during her focused history and examination. Therefore, reflex catecholamine surge to counteract bradycardia may have set the scene for TC evolution in this patient making SSS the most plausible trigger. Also, after the resolution of TC, our patient had episodes of bradycardia requiring cardiac pacing. Thus, TC is unlikely to be the cause of her SSS.

Pacemaker implantation was indicated in our patient due to the documented symptomatic bradycardia [13]. Dual-chamber pacemaker is recommended over single-chamber atrial pacing for all patients with sick sinus syndrome [14]. Also, in our patient, a concomitant atrioventricular nodal disease was expected. Furthermore, cardiac pacing allows adequate rate control (if needed) given that tachycardia-bradycardia syndrome persisted after TC resolution [13]. The patient also had prolongation of the QT interval during the acute phase of TC, which lowers her threshold for the development of torsades de pointes and ventricular fibrillation [2]. In addition, SND in patients with TC with QT prolongation can lead to significant rate variation and consequently higher risk torsades de pointes [3]. Thus, cardiac pacing may potentially reduce the risk of ventricular arrhythmias.

## 4. Conclusion

- Acute symptomatic sick sinus syndrome in patients with preexisting silent sinus node dysfunction can trigger takotsubo cardiomyopathy.
- Understanding precipitating factors of takotsubo cardiomyopathy and identifying the patients at risk of life-threatening arrhythmia can help in refining risk stratification and therapy planning.
- Patients with sick sinus syndrome complicated by takotsubo cardiomyopathy may benefit from pacemaker implantation. However, evaluation on a case-by-case basis is mandatory.
